# General self-efficacy and posttraumatic stress after a natural disaster: a longitudinal study

**DOI:** 10.1186/s40359-016-0119-2

**Published:** 2016-04-06

**Authors:** Egil Nygaard, Ajmal Hussain, Johan Siqveland, Trond Heir

**Affiliations:** Department of Psychology, University of Oslo, Blindern, Postbox 1094, 0317 Oslo, Norway; Center for Child and Adolescent Mental Health, Eastern and Southern Norway (RBUP), Nydalen, Postbox 4623, 0405 Oslo, Norway; Department of Mental Health Services, Akershus University Hospital, Sykehusveien 25, 1478 Lørenskog, Norway; Institute of Clinical Medicine, University of Oslo, Blindern, Postbox 1171, 0318 Oslo, Norway; Norwegian Centre for Violence and Traumatic Stress Studies, Nydalen, Postbox 181, 0409 Oslo, Norway

**Keywords:** Disaster, Posttraumatic stress reactions, PTSD, Self-efficacy

## Abstract

**Background:**

Self-efficacy may be an important factor in individuals’ recovery from posttraumatic stress reactions after a natural disaster. However, few longitudinal studies have investigated whether self-efficacy predicts the course of posttraumatic recovery beyond lower initial levels of distress. The purpose of the present study was to investigate whether general self-efficacy is related to recovery from posttraumatic stress reactions from a longitudinal perspective.

**Methods:**

A total of 617 Norwegians exposed to the 2004 Southeast Asian tsunami completed self-report questionnaires measuring their level of disaster exposure and general self-efficacy at 6 months and posttraumatic stress reactions 6 months and 2 years post-disaster. Predictors of changes in posttraumatic stress reactions were analyzed with multivariate mixed effects models.

**Results:**

Self-efficacy at 6 months post-disaster was unrelated to trauma exposure and inversely related to posttraumatic stress reactions at 6 months and 2 years post-disaster. However, self-efficacy was not related to recovery from posttraumatic stress reactions between 6 months and 2 years post-disaster.

**Conclusions:**

In conclusion, general self-efficacy is related to lower levels of posttraumatic stress reactions in the first months after a disaster but does not appear to be related to improved recovery rates over the longer term.

## Background

Survivors of a natural disaster commonly display posttraumatic stress reactions shortly after the disaster [1–3]. However, over the longer term, most survivors recover from their initial distress [4]. Greater knowledge about the factors that promote this recovery process is highly useful in planning psychosocial disaster interventions. One potentially important factor that promotes recovery is general self-efficacy (GSE), which is broadly defined as individuals’ perceived ability to achieve a desired outcome [5]. Self-efficacy may foster recovery from stress reactions because persons with high levels of self-efficacy use more active and adaptive coping strategies and do not succumb to catastrophizing or other dysfunctional thought patterns [6]. Cross-sectional studies of posttraumatic stress and GSE have shown that GSE is moderately to strongly negatively related to posttraumatic stress reactions after natural disasters [7–9].

Few longitudinal studies have investigated GSE and posttraumatic recovery after disasters, and even fewer studies have controlled for initial levels of posttraumatic stress reactions. Furthermore, the existing studies report somewhat mixed findings regarding the role that GSE plays in recovery from disaster after controlling for initial levels of stress reactions. For example, Wadsworth, Santiago [10] did not control for prior levels of distress; rather, they used longitudinal data to define four trajectory groups. They found that higher levels of perceived coping efficacy among trauma victims were related to more transient posttraumatic stress reactions 6 and 12 months after a hurricane. Benight and Harper [11] found that coping self-efficacy (CSE) 3 to 8 weeks and 1 year after disasters was significantly related to posttraumatic stress reactions 1 year after disasters, even after controlling for acute stress reactions and posttraumatic stress reactions measured at the first assessment. They also found that CSE mediated the relationship between acute distress and later posttraumatic stress reactions. Furthermore, in a study conducted after a natural disaster, they found a similar relationship between CSE and later distress, as assessed by a measure of global psychological distress [12]. A recently published study [13] found that GSE was not significantly related to posttraumatic stress reactions 3 years post-earthquake when posttraumatic stress reactions 1–6 months post-disaster were taken into account. Unfortunately, GSE was assessed at follow-up but not prior to the period of change. Thus, whether GSE predicts the future course of posttraumatic recovery beyond lower initial levels of distress remains unknown.

In the present study, we investigated whether GSE is negatively related to trauma exposure. In general, GSE is highly stable, with trait-like qualities, and may be linked to genetic predispositions [14]; however, traumatic events may alter psychological traits that are normally stable. A recent investigation [15] found that severe traumatic exposure can alter the personality trait of neuroticism – at least temporarily. Therefore, GSE may also change following exposure to trauma and may be negatively related to disaster exposure. This finding could change our understanding of the relationship between GSE and posttraumatic stress reactions. Higher levels of GSE post-disaster may serve as a proxy for lower levels of disaster exposure and, therefore, be related to lowered distress rather than playing a causal role in promoting recovery from stress reactions.

One last issue regarding GSE and posttraumatic stress reactions that interested us was whether the relationship between GSE and posttraumatic stress reactions is linear. We were interested in investigating whether some of the highest levels of perceived GSE might be a representation of denial-based inefficient coping attempts, which we would expect to be related to higher levels of stress reactions. This research question was largely exploratory in nature, but denial as an inefficient coping attempt has been previously described in the literature [16]. However, to our knowledge, whether denial could represent unrealistic beliefs about self-efficacy in some cases has not been previously investigated.

The present study examined GSE and posttraumatic recovery between 6 months and 2 years post-disaster in a large group of Norwegian tourists repatriated after the 2004 Southeast Asia tsunami. We hypothesized that GSE would be unrelated to disaster exposure and that GSE at 6 months would be negatively related to concurrent posttraumatic stress reactions. We also hypothesized that after controlling for initial posttraumatic stress reactions, higher levels of self-efficacy at 6 months post-disaster would be related to improved recovery from posttraumatic stress reactions between 6 months and 2 years post-disaster.

## Methods

### Participants and design

Norwegian police registered the names, personal identification numbers, and addresses of the Norwegian citizens who had been in Southeast Asia during the 2004 tsunami. With permission from the Norwegian Data Inspectorate, this information was made available for our study. A total of 2468 individuals 18 years or older who had been in disaster-affected areas were invited to participate via a postal questionnaire at six (T1) and 24 (T2) months after the tsunami. A total of 868 (35.2 %) and 1170 (47.4 %) participants responded at T1 and T2, respectively, and 657 responded at both time points. Forty participants were excluded due to missing data; thus, the present study included 617 (25.0 %) participants. Of this sample, 213 (34.5 %) participants shared a household with other participants: 94 households included two participants, seven households included three participants and one household included four participants. Additional information about the participants is presented in Table 1.

**Table 1:** Descriptive statistics of the major study variables (*N* = 617)

	*N* (%)/*M* (*SD*)
Gender:	
Male (=0)	276 (44.7 %)
Female (=1)	341 (55.3 %)
Age	43.24 (12.82)
Education^a^:	
Below upper secondary	46 (8.0 %)
Upper secondary	170 (29.6 %)
Up to 4 years of higher education	200 (34.8 %)
More than 4 years of higher education	159 (27.7 %)
Exposure severity:	
Indirectly exposed (=0)	115 (18.6 %)
Exposed but not in danger (=1)	278 (45.1 %)
In danger (=2)	224 (36.3 %)
Perceived threat of death	1.49 (1.42)
Loss of family or friends:	
No (=0)	564 (91.4 %)
Yes (=1)	53 (8.6 %)
Social support satisfaction	5.53 (1.58)
IES-R T1	1.13 (0.83)
IES-R T2	1.07 (0.83)
IES-R change (T2-T1)	−0.07 (0.57)
GPSES T1	3.15 (0.46)

*GPSES* General Perceived Self-Efficacy Scale, *IES-R* Impact of Event Scale-Revised, *T1* 6 months post-disaster; and *T2* 2 years post-disaster

^a^Missing information about education for 42 participants; thus, *n* = 575

The participants in the final sample (*n* = 617) did not differ significantly from those who were not included in the analyses (either because they participated only at one time point (*n* = 724) or were excluded due to missing data (*n* = 40)) with respect to the levels of posttraumatic stress reactions at T1, self-efficacy at T1, changes in posttraumatic stress reactions from T1 to T2, loss, education, or number of participants in the household. However, compared with the participants who were not included, the included participants were generally older (*M*_included_ = 43.2 years, *SD* = 12.8, *n* = 617; *M*_not included_ = 41.4 years, *SD* = 12.9, *n* = 758; *df* = 1373; *p* = .009), had higher levels of posttraumatic stress reactions at T2 (*M*_included_ = 1.1, *SD* = 0.8, *n* = 616; *M*_not included_ = 0.9, *SD* = 0.8, *n* = 551; *df* = 1165; *p* < .001), were more likely to be women (57.2 % of *n*_*included*_ = 617 vs. 48.2 % of *n*_*not included*_ = 758; chi square (1) = 11.2; *p* = .001) and were exposed to greater danger (36.3 % of *n*_*included*_ = 617 in danger vs. 26.9 % of *n*_*not included*_ = 238; chi square (2) = 11.2, *p* = .004).

Earlier published studies of the Norwegians who experienced the 2004 tsunami have evaluated the representativeness of responders relative to non-responders through telephone interviews with non-responders [17] and through register data [18]. They documented that responders at T1 were more likely than non-responders to have had more serious exposure [17] and to be women [18], but these two groups were of similar age [18]. Responders at T1 were similar to the Norwegian age- and gender-controlled population with regard to employment and marital status but had higher education levels, on average [18].

### Ethics and consent

The Norwegian Data Inspectorate (project: 12858) approved the project. Participants provided their written informed consent through the questionnaire.

### Measures

#### Posttraumatic stress reactions

Posttraumatic stress reactions were measured with the Impact of Event Scale-Revised (IES-R) [19] at 6 months and 2 years post-disaster. The IES-R consists of 22 items, with five response alternatives, related to the degree of distress intensity during the previous 2 weeks (“Not at all” (0), “A little” (1), “Moderately” (2), “Quite a bit” (3), and “Extremely” (4)). The total mean score was calculated based on all items. The psychometric properties of the IES-R have been extensively evaluated and deemed acceptable [20]. The IES-R has demonstrated acceptable reliability in a Norwegian nonclinical sample [21]. Furthermore, the internal consistency of the IES-R in the present sample was high (Cronbach’s α = 0.96 and 0.97 at T1 and T2, respectively).

#### General self-efficacy

At 6 months post-disaster, the participants responded to the Norwegian version of the General Perceived Self-Efficacy Scale (GPSES) [22, 23] as a measure of GSE. The GPSES measures the participants’ confidence in their ability to control challenging environmental demands by performing adaptive actions. The GPSES consists of 10 statements about mastery with four response alternatives: “Not at all true” (1), “Hardly true” (2), “Moderately true” (3), and “Exactly true” (4). The total mean score is calculated based on all items. The GPSES is the most frequently used scale for measuring perceived self-efficacy, and it has been found to have good psychometric qualities [24]. The internal consistency of the GPSES in the present sample was good (Cronbach’s α = 0.90).

#### Exposure

At the 6 month assessment, the participants reported their traumatic exposure during the tsunami, such as whether they were caught, touched or chased by waves; whether they suffered physical injuries; whether they witnessed the death and suffering of others; whether they were uncertain of the fate of family members or close friends; and whether their close relative or friend died [25]. The participants were divided into three groups based on the severity of their exposure: a “danger exposed” group consisting of individuals caught, touched or chased by waves; a “non-danger exposed” group directly exposed to the disaster (suffered physical injury, witnessed the death and suffering of others, faced uncertainty regarding the fate of family members or close friends, or had a close relative or friend die) but not exposed to immediate life-threatening situations by the waves; and an “indirectly exposed” group consisting of participants who had been present in Southeast Asia at the time of the tsunami but who had not been exposed to any of the situations described above [26]. Both the danger exposed group and the non-danger exposed group were considered to meet the DSM-IV criteria for a traumatic stressor. Still, we assumed that the danger exposed more likely had been present in the epicenter of the disaster, and thus, we chose to use two categories of directly exposed respondents. Previously, we have shown that division by severity of exposure to danger was closely related to health outcomes [26].

#### Perceived threat of death

At 6 months post-disaster, the participants reported their self-perceived threat of death during the disaster. The question had five possible response alternatives: “None” (0), “Small” (1), “Moderate” (2), “Major” (3), and “Overwhelming” (4).

#### Social support

At the 6 month assessment, satisfaction with social support was assessed with one question: “All in all, are you satisfied with the social support you received after the disaster?” The participants responded on a 7-point Likert scale ranging from “Absolutely not” (1) to “Yes, very” (7).

### Data analysis

Participants with unknown exposure levels and participants with missing responses to more than four items on the IES-R or the GPSES were excluded from the analysis. This limit was set because the participants either missed a few (four or less) or most items. The procedure for excluding cases with substantial missing data was determined in advance based on previous procedures [27]. For the remaining participants, missing values were not missing completely at random (*p* ≤ .01 on Little’s test). Thus, missing values for posttraumatic stress reactions, GPSES, social support and perceived threat of death were replaced with 20 imputations determined by using an iterative Markov chain Monte Carlo method (fully conditional specification).

A bivariate overview of all relationships was performed with Pearson’s correlation, and the bivariate mean differences between multiple groups were assessed with one-way ANOVA. Because study participants were partly clustered together in families with shared households, we applied mixed effects linear regression analyses [28]. All multiple mixed effects models controlled for gender, age, exposure, perceived threat of death, loss and social support. The curvilinear relationships between GSE and changes in stress reactions were investigated by dividing stress reactions into quartiles and by analyzing the quadratic relationships between GPSES score and changes in posttraumatic stress reactions. All continuous variables were standardized before they were entered into the regression models. All analyses were conducted using the statistical package IBM SPSS Statistics, version 21, and the significance level was set at 0.05.

## Results

### Bivariate correlations and the relationship between exposure and general self-efficacy

A correlation matrix of all variables is presented in Table 2. Females had significantly higher levels of posttraumatic stress reactions at both measurement times, but there was no significant relationship between gender and GPSES scores. Age was not significantly related to the level of posttraumatic stress reactions or GPSES. Social support was significantly related to less severe posttraumatic stress reactions and higher GPSES scores. Levels of posttraumatic stress reactions at T1 and T2 were highly correlated. Lower scores on GPSES were significantly related to high levels of posttraumatic stress reactions both at T1 (b^*^ =−0.29, 95 % CI−0.36,−0.22, *p* ≤ 0.001) and at T2 (b^*^ =−0.26, 95 % CI−0.33,−0.18, *p* ≤ 0.001) but not related to changes in posttraumatic stress reactions (b^*^ =−0.06, 95 % CI−0.14, 0.02, *p* = 0.14). Only gender (with females having the greatest reductions) and levels of posttraumatic stress reactions at either time point were significantly correlated with changes in posttraumatic stress reactions. Whereas a decrease in posttraumatic stress reaction was related to higher levels of stress reactions at T1 (*r* =−0.33), it was related to lower levels of stress reactions at T2 (*r* = 0.35).

**Table 2:** Correlation matrix of the major study variables (*N* = 617)

	1	2	3	4	5	6	7	8	9
1 Gender:									
Male (=0)									
Female (=1)									
2 Age	−0.16***								
3 Exposure severity:	0.04	−0.08*							
Indirectly exposed (=0)									
Exposed but not in danger (=1)									
In danger (=2)									
4 Perceived threat of death	0.04	−0.04	0.65***						
5 Loss of family or friends:	−0.04	−0.02	0.17***	0.23***					
No (=0)									
Yes (=1)									
6 Social support satisfaction	0.14***	−0.12**	−0.11*	−0.13**	−0.09*				
7 IES-R T1	0.18***	0.01	0.41***	0.47***	0.22***	−0.36***			
8 IES-R T2	0.10*	0.03	0.37***	0.42***	0.20***	−0.32***	0.77***		
9 IES-R change (T2-T1)	−0.11**	0.03	−0.05	−0.06	−0.04	0.05	−0.33***	0.35***	
10 GPSES T1	−0.07	−0.06	−0.08	−0.08*	−0.03	0.24***	−0.30***	−0.26***	0.07

Correlations are based on multiple imputed data

*GPSES* General Perceived Self-Efficacy Scale, *IES-R* Impact of Event Scale-Revised, *T1* 6 months post-disaster; and *T2* 2 years post-disaster

*p ≤ .05; **p ≤ .01; and ***p ≤ .001

As expected, all measures of exposure were highly related to higher levels of posttraumatic stress reactions. However, only perceived threat of death was significantly correlated with GPSES, with higher perceived threat related to lower scores on GPSES. A one-way ANOVA analyzing group differences showed that the three groups of exposure severity showed significant differences in the level of posttraumatic stress reactions at both time points (*F* = 62.67 at T1 and *F* = 49.65 at T2, both with *p* < .001), but exposure severity was not significantly related to the GPSES scores. GPSES was also not related to disaster exposure severity when analyzed with a mixed effects model with family as the multilevel subject (*M*_indirectly exposed_ = 3.2, *SD* = 0.5; *M*_exposed non-danger_ = 3.2, *SD* = 0.3; *M*_in danger_ = 3.1, *SD* = 0.3; b* = 0.08, *p* = .15 for indirect vs. in danger and b* = 0.07, *p* = .10 for exposed non-danger vs. in danger).

### The relationship between general self-efficacy and posttraumatic stress reactions at 6 months post-disaster

Multiple mixed effects linear regression analyses were conducted to investigate whether GPSES scores contributed to explaining levels of posttraumatic stress reactions at 6 months post-disaster. Two models were run, both with and without GPSES as a predictor (Table 3). GPSES was significantly related to the level of posttraumatic stress reactions at 6 months after gender, age, exposure, perceived threat of death, loss and social support were controlled, and it contributed to explaining an additional 3.2 % of the variance in the level of posttraumatic stress reactions.

**Table 3:** Predictions of posttraumatic stress reactions at 6 months (*N* = 617)

	Multiple analyses model 1	Multiple analyses model 2 (model 1 + GPSES)
	IES-R at 6 months	IES-R at 6 months
Fixed effects:		
Intercept	0.63 (0.38, 0.89)***	0.61 (0.36, 0.86)***
Gender		
Male	−0.46 (−0.58,–0.35)***	−0.42 (−0.53,−0.31)***
Female^a^	0	0
Age	0.03 (−0.03, 0.10)	0.03 (−0.04, 0.09)
Exposure		
Indirectly exposed	−0.44 (−0.68,−0.20)***	−0.44 (−0.67,−0.21)***
Exposed but not in danger	−0.05 (−0.23, 0.14)	−0.03 (−0.21, 0.14)
In danger^a^	0	0
Perceived threat of death	0.32 (0.23, 0.41)***	0.32 (0.23, 0.40)***
Loss		
No loss	−0.35 (−0.58,−0.12)**	−0.35 (−0.57,−0.12)**
Loss of family or close friend^a^	0	0
Social support satisfaction	−0.32 (−0.38,−0.26)***	−0.28 (−0.34,−0.22)***
GPSES		−0.18 (−0.24,−0.12)***
Explained variance:		
Between households	38.2 %	42.9 %
Between individuals within households	36.9 %	39.6 %
Total explained variance	37.6 %	40.8 %
Model fit:		
AIC (original data)	1287.96	1267.34

Multilevel linear regression analyses controlled for the effect of the same address based on multiple imputed data. The values are regression coefficients (with 95 % confidence intervals presented in parentheses). All continuous variables were standardized (*M* = 0, *SD* = 1) before being entered into the model as dependent or independent variables. All predictors were measured at 6 months post-disaster

*AIC* Akaike’s Information Criterion, *GPSES* General Perceived Self-Efficacy Scale, *IES-R* Impact of Event Scale-Revised

**p ≤ .01; ***p ≤ .001

^a^Females, those who had been exposed to danger, and those who had lost family or close friends were set to have a mean of 0 in the mixed effects models

### The relationship between general self-efficacy and recovery from posttraumatic stress reactions between 6 months and 2 years post-disaster

Similar multiple mixed effects linear regression analyses were performed to assess the relationship between GPSES scores at 6 months and changes in posttraumatic stress reactions between 6 months and 2 years post-disaster (Table 4). In these analyses, GPSES did not explain the changes in posttraumatic stress reactions from 6 months to 2 years post-disaster after levels of posttraumatic stress reactions at 6 months were taken into account. Thus, GPSES scores at 6 months were related to posttraumatic stress reactions at 6 months but not to changes in posttraumatic stress reactions between 6 months and 2 years.

**Table 4:** Predictions of changes in posttraumatic stress reactions (*N* = 617)

	Multiple analyses model 1	Multiple analyses model 2 (model 1 + GPSES)
	Change in IES-R (2 years – 6 months)	Change in IES-R (2 years – 6 months)
Fixed effects:		
Intercept	0.08 (−0.23, 0.39)	0.08 (−0.23, 0.39)
Gender		
Male	0.05 (−0.11, 0.20)	0.05 (−0.10, 0.20)
Female^a^	0	0
Age	0.03 (−0.04, 0.11)	0.03 (−0.05, 0.11)
Exposure		
Indirectly exposed	−0.14 (−0.42, 0.14)	−0.14 (−0.42, 0.14)
Exposed but not in danger	0.00 (−0.21, 0.21)	0.00 (−0.21, 0.22)
In danger^a^	0	0
Perceived threat of death	0.11 (−0.00, 0.22)	0.11 (0.00, 0.22)*
Loss		
No loss	−0.08 (−0.36, 0.20)	−0.08 (−0.36, 0.19)
Loss of family or close friend^a^	0	0
Social support	−0.07 (−0.16, 0.02)	−0.07 (−0.15, 0.02)
IES-R at 6 months	−0.43 (−0.52,−0.33)***	−0.44 (−0.54,−0.34)***
GPSES		−0.05 (−0.13, 0.03)
Explained variance:		
Between households	15.9 %	14.5 %
Between individuals within households	10.8 %	11.3 %
Total explained variance	12.0 %	12.0 %
Model fit:		
AIC (original data)	1481.02	1485.12

Multilevel linear regression analyses controlled for the effect of a mutual address based on multiple imputed data. The values are regression coefficients (95 % confidence intervals in parentheses). All continuous variables were standardized (*M* = 0, *SD* = 1) before being entered into the model as dependent or independent variables. All predictors were measured at 6 months post-tsunami

*AIC* Akaike’s Information Criterion, *GPSES* General Perceived Self-Efficacy Scale, *IES-R* Impact of Event Scale-Revised

*p ≤ .05 and ***p ≤ .001

^a^Females, those who had been exposed to danger, and those who had lost family or close friends were set to have a mean of 0 in the mixed effects models

The mixed effects models were rerun with only participants who had scored above two on their level of posttraumatic stress reactions at T1 (*n* = 100) to control for whether the missing relationships between GPSES and changes in IES-R scores were related to a floor effect. There were no significant relationships between GPSES and changes in IES-R scores.

Because no linear relationship was found, GPSES was divided into quartiles in a post hoc analysis to investigate a possible curvilinear relationship between self-efficacy and changes in posttraumatic stress reactions. We expected GPSES scores in the middle range to be most highly related to recovery from posttraumatic stress reactions. This hypothesis was based on the notion that the highest level of self-efficacy might be related to a self-enhancing and unrealistic self-image as part of a self-denial mode of coping. The relationships between GPSES and posttraumatic stress reactions at each time point were linear, with each quartile displaying a lower level of posttraumatic stress reactions than the previous quartile (Table 5). There were no significant differences between the four quartiles with respect to changes in posttraumatic stress reactions.

**Table 5:** The relationships between quartile-divided general self-efficacy and posttraumatic stress reactions

	IES-R at 6 months Mean (SD)	IES-R at 2 years Mean (SD)	Changes in IES-R (2 years – 6 months) Mean (SD)^a^
1st quartile GPSES (*n* = 130)	1.48 (0.96)	1.33 (0.94)	−0.15 (0.68)
2nd quartile GPSES (*n* = 166)	1.22 (0.79)	1.21 (0.79)	−0.01 (0.58)
3rd quartile GPSES (*n* = 156)	1.06 (0.74)	1.01 (0.77)	−0.05 (0.50)
4th quartile GPSES (*n* = 165)	0.85 (0.74)	0.77 (0.72)	−0.08 (0.51)
Sign test	*F* (3, 613) = 15.85, *p* < .001	*F* (3, 613) = 13.56, *p* < .001	*F* (3, 613) = 1.57, *p* = .20
Post hoc	1 > 2, 3 and 4	1 > 3 and 4	None
	2 > 4	2 and 3 > 4	

Significant differences between quartiles were tested with one-way ANOVAs based on multiple imputed data. The Bonferroni test was used post hoc to investigate which quartiles were significantly different from each other

*GPSES* General Perceived Self-Efficacy Scale, *IES-R* Impact of Event Scale-Revised

^a^Negative figures indicate a decrease in posttraumatic stress reactions from 6 months to 2 years post-disaster

**Fig. 1:**
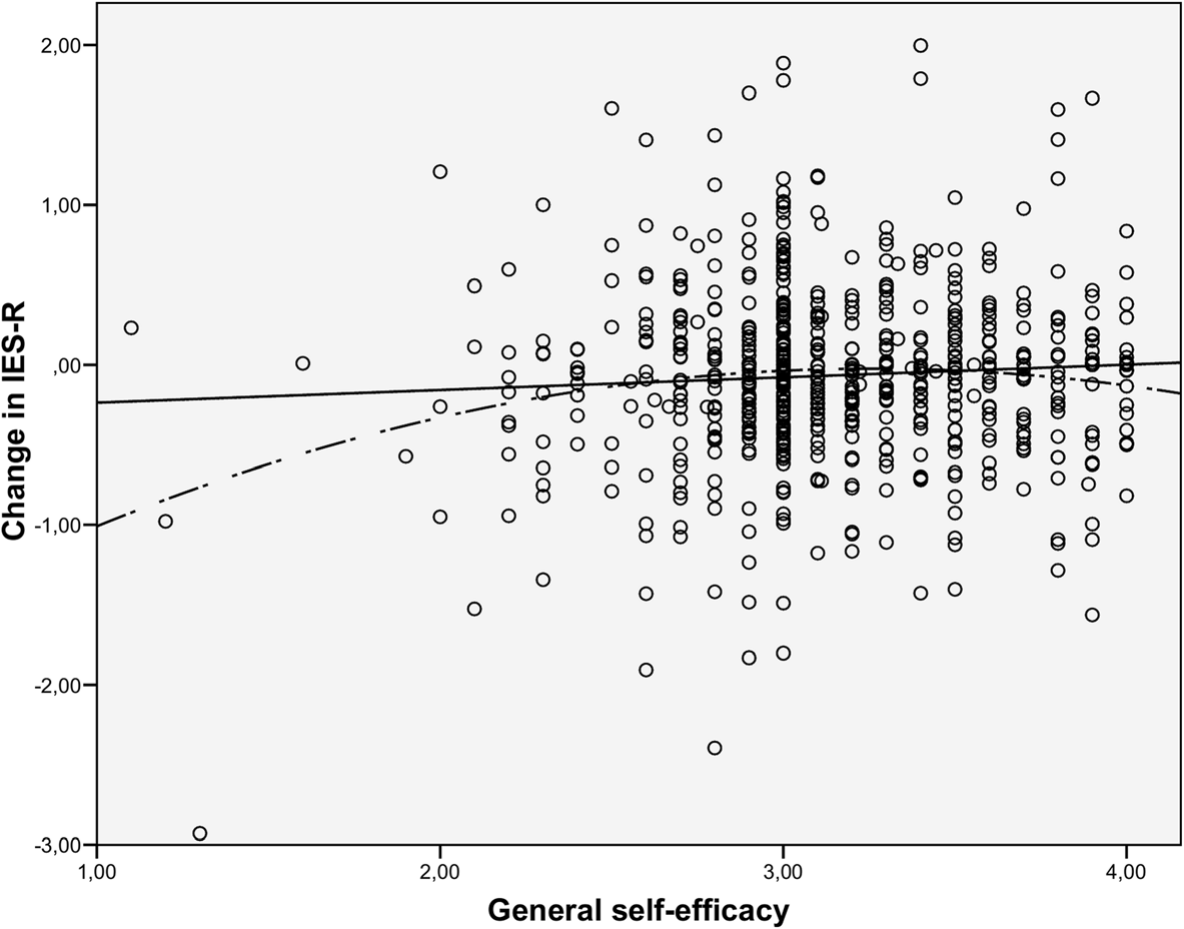
General self-efficacy and recovery from posttraumatic stress reactions. The bivariate relationships between general perceived self-efficacy and changes in posttraumatic stress reactions (Impact of Event Scale-Revised). Both linear and quadratic regression lines are included

The bivariate relationship between GPSES and changes in posttraumatic stress reactions is further presented in a scatterplot in Fig. 1. Although the linear relationship was nonsignificant (*F*(1, 615) = 2.51, *p* = .11), there was a statistically significant quadratic relationship between GPSES and posttraumatic stress reactions (*F*(2, 614) = 5.74, *p* = .003). However, this curvilinear relationship was in the opposite direction of the hypothesized relationship, and the relationship is difficult to discern visually. The curvilinear relationship in Fig. 1 may have been observed because of outliers. The quartile division in Table 5, which is less sensitive to outliers, did not show such a curvilinear relationship.

The relationship between quartile-divided GPSES and changes in posttraumatic stress reactions was tested in a multiple mixed effects model similar to the second model presented in Table 4 to examine whether a non-linear relationship is observed after the covariates are included. The total effect of GPSES still did not significantly contribute to explaining changes in IES-R scores from 6 months to 2 years post-disaster (*F* (3, 595) = 2.03, *p* = .11). Additionally, the model showed the same curvilinear relationship between GPSES and changes in IES-R scores as indicated in Table 5 and Fig. 1; thus, the group in the second quartile of GPSES showed the least degree of change in IES-R scores after covariates were controlled.

## Discussion

We found that higher levels of general perceived self-efficacy were related to lower levels of posttraumatic stress reactions at both 6 months (T1) and 2 years (T2) post-disaster. However, GSE did not have a linear or curvilinear relationship with recovery from posttraumatic stress reactions between 6 months and 2 years post-disaster. GPSES scores were negatively correlated with trauma exposure severity, but the correlation was nonsignificant. Furthermore, GPSES displayed only a marginally statistically significant negative correlation with perceived threat.

The finding of an association between self-efficacy and lower levels of distress in the aftermath of a natural disaster is congruent with the previous findings of some cross-sectional studies [29, 30] and a few longitudinal studies [11, 12]. The findings are also congruent with research on other types of collective trauma, such as war and terrorist attacks [31] and individual trauma [32]. However, the correlations between GSE and posttraumatic stress reactions found in the present study (*r* =−0.30 and−0.26 at 6 months and 2 years post-disaster, respectively) are lower than those found in a systematic review of longitudinal studies on collective traumas (Luszczynska, Benight [31] (weighted *r* values between −0.55 and −0.62). These differences may be related to our use of a general, rather than a trauma coping-specific, measure of self-efficacy. However, our findings support the idea that core beliefs about the ability to master challenging environmental demands promote resilience to adversity and effective coping [6]. Nevertheless, the length of time that self-efficacy aids the recovery process after a disaster remains uncertain.

Because our first assessment of posttraumatic stress reactions was 6 months post-tsunami, the current study could not separate trajectories of resilience against developing posttraumatic stress reactions from trajectories of recovery from posttraumatic stress reactions prior to 6 months post-disaster. However, GSE failed to predict recovery between 6 months and 2 years post-disaster when the initial level of posttraumatic stress reactions was controlled. This finding is contrary to the findings of two of the few longitudinal disaster studies that also controlled for distress levels at T1 [11, 12]. In these studies of natural disaster survivors, Benight and colleagues found that CSE predicted psychological distress 8–12 months post-disaster after controlling for initial distress levels 1–4 months post-disaster. The difference in findings between prior studies and the present study may be due to several differences in the study designs and post-disaster settings. Specifically, Benight and colleagues investigated participants living in the disaster area, while the majority of our respondents escaped secondary stressors because they were evacuated to Norway shortly after the disaster. Benight, Ironson [12] measured the outcome of general distress, while we measured posttraumatic stress reactions. Lastly, they studied the more specific concept of CSE, while we measured the role of the more general concept of GSE. In addition, Benight and colleagues examined individuals’ acute disaster responses (1–4 months post-disaster) and medium-range disaster recovery (8–12 months); thus, their period of examination differs from our period. Our findings of a lack of relationship with recovery are, however, similar to the findings of Warner, Gutierrez-Dona [13]. These authors found that posttraumatic stress reactions 3 years after an earthquake were not significantly related to concurrent GSE after posttraumatic stress reactions at 1 to 6 months were taken into consideration. Their study was nearly identical to our study in regards to timeframe, type of disaster, assessment tools and type of analyses. However, there were some differences in the studies. For example, in their study, the participants lived in the disaster area and GSE was assessed at follow-up. Lastly, contrary to our study, the study conducted by Wadsworth, Santiago [10] reported that participants who were resilient displayed higher levels of efficacy than those who had chronic stress reactions 1 year after a hurricane.

Our findings suggest that the estimated effect of self-efficacy on posttraumatic recovery is reduced when baseline levels of posttraumatic stress reactions are adequately adjusted and when a longitudinal design is used rather than a cross-sectional design [33]. Thus, our finding of a positive relationship between GSE and posttraumatic stress reactions at 6 months but not at 2 years post-disaster after controlling for stress reactions at 6 months is in line with a recent meta-analysis that indicated that the frequently observed positive relationship between self-efficacy and performance is partly a product of past performance [34]. Furthermore, our finding that the participants’ GSE levels were not substantially related to their level of disaster exposure supports the assumption that GSE is rather stable and may have trait-like qualities [35, 36]. Thus, the finding that exposure is highly related to posttraumatic stress reactions but not to GSE supports our interpretation regarding the causal direction of the relationship between GSE and posttraumatic stress reactions: GSE influences the level of posttraumatic stress reactions, whereas the level of posttraumatic stress reactions does not influence GSE. This interpretation is also supported by the results of a large cross-lagged study on the relationship between CSE and stress reactions after a wide range of traumatic exposures. This study found that prior CSE predicted later stress reactions but did not find that prior stress reactions predicted later CSE [32].

### Methodological considerations

The strength of the current study is that we were able to follow a relatively large population after a global disaster. Our finding was replicated in the subgroup with high initial levels of posttraumatic stress reactions and, therefore, cannot be explained as a statistical artifact resulting from a floor effect. In addition, the results remained unchanged even after we controlled for important and familiar risk factors for posttraumatic stress reactions, including perceived threat of death, social support [37], direct exposure and loss.

Nearly all Norwegians who evacuated from the disaster area were invited to participate, reducing sample selection bias. The participants were similar to the age- and gender-adjusted Norwegian population with regard to employment and marital status but had higher education levels than the Norwegian population and were more often women than the nonparticipants [18]. Such skewness may influence the generalizability of the study.

Limitations of our study include the relatively low response rate. Given both the directionality of the participation vs non-responders [17] and dropout bias, the included participants seem to represent the most heavily exposed Norwegian tourists in the disaster-stricken areas. Our findings are also limited by the use of self-reports and our reliance on single instruments to assess GSE and posttraumatic stress reactions. Social support after the disaster until the assessment at 6 months post-tsunami was measured with one question. Although single-item measures of social support have been found to be valid and reliable [38], they do not take into account intrapersonal variations such as changes over time and the provider of social support.

Furthermore, the study did not include any measurements prior to 6 months post-tsunami. Thus, we cannot rule out the possibility that relationships between exposure, posttraumatic stress reactions and GSE, which are not reflected in the present study, existed before this point. For example, we cannot determine whether GSE is a protective or recovery factor from stress reactions before 6 months post-tsunami. We also cannot rule out the possibility that a significant relationship existed between exposure and GSE in the immediate aftermath of the disaster but disappeared at 6 months. However, both exposure and GSE are considered to be stable factors; thus, the relationship between these factors should be quite similar at 6 months and before this time point. Moreover, we cannot determine whether the relationship between GSE and posttraumatic stress reactions is due to a causal mechanism or spurious effects from other non-measured factors. A related problem is that the perception of threat may have changed over time [18]. Thus, it would have been preferable if perceived threat was measured soon after the event.

GSE, which reflects a generalization of self-efficacy across various domains of functioning, may be less suitable for capturing self-efficacy after disaster than CSE. This might be an important issue because it has been suggested that perceived self-efficacy should be conceptualized and measured in a situation-specific manner [39]. Thus, most research on self-efficacy and recovery after disasters has focused on CSE – “the perceived capability to manage one’s personal functioning and the myriad environmental demands of the aftermath occasioned by a traumatic event” [6]. Nevertheless, persons who experience a disaster and subsequent posttraumatic stress reactions must adjust their lives to multiple demands rather than to a specific task. For example, challenges such as repairing material damages and living in interrupted societies are less appropriate when the entire disaster population is repatriated to a non-affected home country. In addition, although the concept of CSE may more precisely capture the cognitive and intrapersonal processes relevant for post-disaster coping and recovery, in some cases, this measurement approach may be semantically too close to posttraumatic stress reactions. Whereas some authors seem to have succeeded in avoiding an overlap between CSE and PTSD (for example, the measure of CSE used by Benight, Ironson [40] after Hurricane Andrew and Hurricane Opal), other authors have measured CSE with items that overlap with the diagnostic symptoms of PTSD. For example, Sumer, Karanci [29] used a CSE scale with four items, including “I’m able to think about the earthquake and those I lost more comfortably,” which is very similar to, but with opposite directionality, measures of PTSD. For example, the Impact of Event scale, which Sumer, Karanci [29] used to measure PTSD, includes the items “I tried not to think about it” and “Any reminder brought back feelings about it.” In other words, some symptoms and troubles commonly experienced after a disaster are similarly measured and are simply worded in reverse as compared with measures of CSE.

## Conclusions

Our findings indicate that self-efficacy is related to disaster survivors’ successful coping with post-disaster adversities. However, the positive effect of self-efficacy in promoting coping seems to be time limited, with the strongest effects occurring during the first months post-disaster. It is debatable whether our results are applicable to clinical settings in which therapists aim to reverse people’s negative views regarding their ability to overcome adversity [41]. Self-efficacy, as a useful post-disaster intervention target, is mostly based on social cognition theory [6] and has received some empirical support from trauma research [24, 42]. However, it is acknowledged that the current understanding is not based on evidence from experimental studies [31]. Furthermore, it has also been argued that belief in one’s capabilities may be self-debilitating [43, 44] and that perceived self-efficacy fails to predict future performance [34, 45]. Our findings support such critical stances only in part and, instead, support the notion that GSE may have a positive effect on people who have experienced disasters, particularly in the first months post-disaster. Whether GSE provides protection against the development of chronic mental health problems in the aftermath of traumatic experiences – or whether it aids in the recovery from such problems – requires further investigation.

### Availability of data and materials

Public availability of data would compromise the respondents’ privacy. According to the approval from The Norwegian Data Inspectorate, the data are to be stored properly and in line with the Norwegian Law of privacy protection. However, anonymized data are freely available to interested researchers upon request, pending ethical approval from the Ethics committee. Interested researchers can contact project leader Prof. Trond Heir (trond.heir@medisin.no) with requests for the data underlying these findings.

## Abbreviations

CSE: coping self-efficacy
GPSES: general perceived self-efficacy scale
GSE: general self-efficacy
IES-R: impact of event scale-revised
